# Down syndrome associated childhood myeloid leukemia with yet unreported acquired chromosomal abnormalities and a new potential adverse marker: dup(1)(q25q44)

**DOI:** 10.1186/s13039-018-0370-8

**Published:** 2018-03-13

**Authors:** Faten Moassass, Abdulsamad Wafa, Thomas Liehr, Ayman Al-Ablog, Walid AL Achkar

**Affiliations:** 1 Molecular Biology and Biotechnology Department, Human Genetics Division, Chromosomes Laboratory, Atomic Energy Commission of Syria, P.O. Box 6091, Damascus, Syria; 2 Jena University Hospital, Institute of Human Genetics, Am Klinikum 1, 07747 Jena, Germany

**Keywords:** Down syndrome, Trisomy 21, AML, Acquired chromosomal abnormalities (ACAs), Clone evolution, Cytogenetics, Fluorescence in situ hybridization (FISH), Prognostic factors

## Abstract

**Background:**

Children with constitutional trisomy 21, i.e. Down syndrome (DS, OMIM #190685) have a 10 to 20-fold increased risk for a hematopoietic malignancy. They may suffer from acute lymphoblastic leukemia or acute myeloid leukemia (AML). AML referred to as myeloid leukemia of Down syndrome (ML-DS) is observed especially after birth at an early gestational age and characterized by enhanced white blood cell count, failure of spontaneous remission, liver fibrosis or liver dysfunction, and is significantly associated with early death. There are only few studies yet focusing on the clonal cytogenetic changes during evolution of ML-DS.

**Case presentation:**

In a 1.4-year-old boy with DS an immunophenotype consistent with AML-M1 according to French-American-British (FAB) classification was diagnoses. Cytogenetic and molecular cytogenetic analyses revealed, besides constitutional free trisomy 21, an unbalanced translocation as der(16)t(1;16)(q25.3;q24), plus a balanced translocation t(3;20)(q25;q13.1). A poor clinical outcome was observed here.

**Conclusions:**

To the best of our knowledge, an ML-DS case associated with identical acquired chromosomal abnormalities was not previously reported. Our findings suggest that especially partial trisomy 1q25 to 1q44 may be indicative for a poor prognosis in ML-DS.

## Background

Children with trisomy 21 or Down syndrome (DS, OMIM #190685) have a compared to normal population 10- to 20-fold increased risk for developing an acute leukemia; lymphoblastic as well as myeloid leukemia were reported [1, 2]. Acute myeloid leukemia- (AML-) affected children develop a unique type of malignancy, referred to as myeloid leukemia of Down Syndrome (ML-DS), which is recognized as a separate entity in the actual World Health Organization (WHO) classification of leukemia [3]. ML-DS is especially found in children born at early gestational age and is characterized by enhanced white blood cell (WBC) count, failure of spontaneous remission, as well as liver fibrosis or liver dysfunction. Also ML-DS is significantly associated with poor outcome and early death [4–6]. ML-DS cases have, according to French-American-British (FAB) classification, in the majority of the cases M7 morphology, thus they are also called acute megakaryoblastic leukemia (AMKL) cases. As most ML-DS cases are young at diagnosis, the disease occurs almost exclusively in children < 5 years old. A beneficial clinical outcome may occur if treated with reduced intensity chemotherapy protocols without stem cell transplantation [7–9].

Even though data on cytogenetics of ML-DS are scarce, it is known that the karyotypic patterns of this entity are different from those observed in AML of children without DS, e.g. translocations t(8;21), t(15;17), t(9;11), inversion inv.(16), as well as AMKL associated translocations t(1;22) and t(1;3) are rather typical for ML-DS [10–12]. The most frequent imbalances in ML-DS are duplications in 1q (16%), or deletions in 7p (10%) and/or 16 (7.4%) [10]. However, the potential clinical impact of these cytogenetic abnormalities is not known, yet. Therefore, the importance of studying and reporting cytogenetic alterations for better classification and risk stratification of ML-DS and non-DS-AML is well recognized [5, 10–14]. There is especially controversial data on the impact of acquired chromosomal abnormalities (ACAs) in ML-DS (see [4, 14] versus [5, 11]).

Here, we describe a typical ML-DS case with two yet unreported ACAs involving chromosomes 1 and 16 as well as 3 and 20, obviously associated with a poor prognosis.

## Case presentation

A 1.4-year-old boy with DS without familial medical history of malignancy presented with 10 days consisting flu and fever, being pallor and unconscious. This patient was the fifth child of healthy, unrelated parents. The mother and the father were at birth of the child, 42 and 54 years old, respectively. Also there was no infection in the pregnant woman during the pregnancy.

At diagnosis the small boy was found to have septicemia, acidosis, dehydration, and lung crackles. Physical examination and ultrasound showed hepatomegaly. His hematological parameters revealed low hemoglobin level (Hgb) (4 g/dl), low platelet count (47 × 10^9^/l), and elevated WBC count. Biochemistry determined urea of 118 mg/dl (normal value up to 40 mg/dl) but normal creatinine levels (0.1 mg/dl). Thus, he was diagnosed as ML-DS patient. The patient received blood transfusion repeatedly, stayed in the hospital for 1 week, and then was transferred to hematological malignancy hospital to confirm diagnosis and treatment. Peripheral blood cell analyses revealed a WBC count of 59.08 × 10^9^/l (18.8% neutrophils, 47.2% lymphocytes, 0.1% eosinophiles, 29.6% monocytes and 4.3% basophiles), red blood cells count of 4.93 × 10^6^/mm^3^, Hgb level of 11.2 g/dl, and platelet count of 24 × 10^9^/l. Blasts in bone marrow aspiration were present in 32% of analyzed cells. The patient had not received any chemotherapy treatment and died unfortunately died 9 days after diagnosis from the disease due to respiratory arrest, and before cytogenetic and flow-cytometric results were available. His mother agreed with scientific evaluation of the case and the study was approved by the ethical committee of the Atomic Energy Commission, Damascus, Syria.

**Fig. 1:**
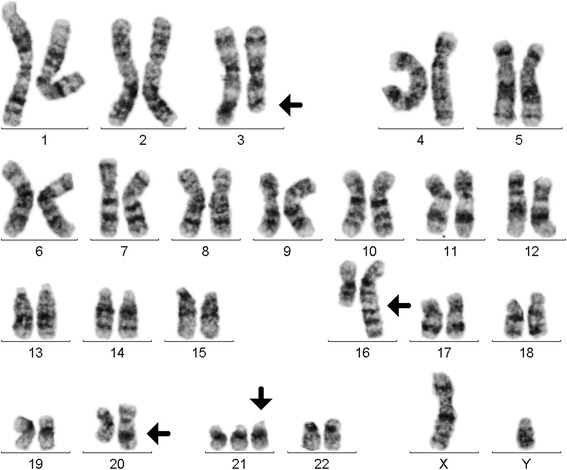
GTG-banding revealed an unbalanced translocation t(1;16)(q25.3;q24) and balanced translocation t(3;20)(q25;q13.1) in 17/20 metaphases. All derivative chromosomes are marked and highlighted by arrow heads

**Fig. 2:**
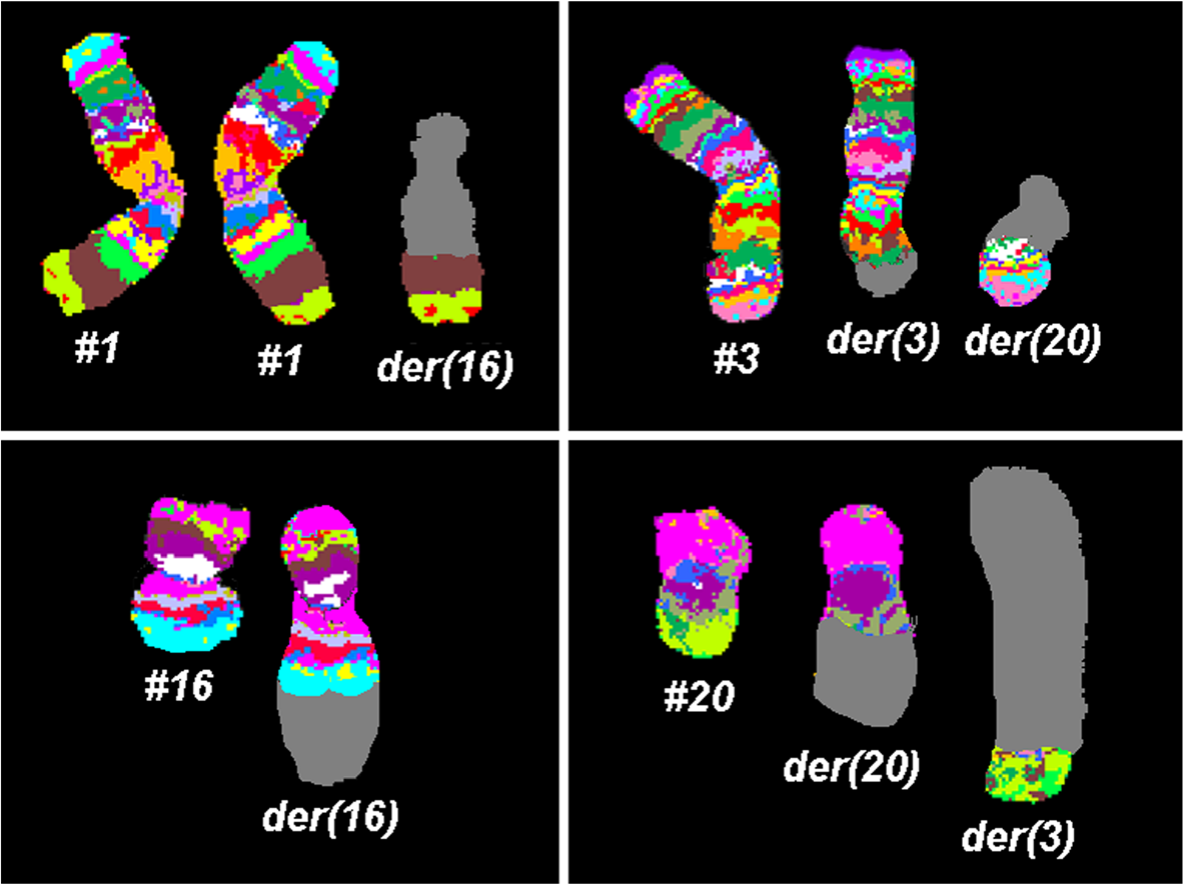
Karyotype and chromosomal aberrations were confirmed using molecular cytogenetic approaches. aMCB results are shown. The normal chromosomes (#) are depicted on the left side of each image and the derivative of the other chromosomes on the right side of normal chromosomes. The unstained regions when suing chromosome-specific aMCB-probesets on the derivative chromosomes are shown in gray. Abbreviations: # = chromosome; der = derivative chromosome

GTG-banding on peripheral blood sample revealed a karyotype of 47,XY,t(1;16)(?;?),t(3;20)(?;?),+21c[17]/47,XY,+21c[3] (Fig. 1). Further studies were performed by molecular cytogenetics (Fig. 2). Dual-color-FISH (D-FISH) using specific WCP probes for chromosomes #1, #3, #16, and #20 confirmed that no other chromosomes were involved besides #1 and #16 in an imbalanced plus #3 and #20 in a balanced translocation (data not shown). aMCB, using probes for chromosomes #1, #3, #16, and #20 (Fig. 2) revealed the following final karyotype:

- 47,XY,der(16)t(1;16)(q25.3;q24),t(3;20)(q25;q13.1),+21c[17]/47,XY,+21c[3]

Immunophenotyping of peripheral blood specimen characterized this case as AML-M1 according to FAB classification. The blast cell population (32% of leukocytes) was positive for CD45^dim^, CD7, CD13dim, CD33, HLA-DR, CD38, and CD117 and negative for CD64, CD34, CD10, CD15, CD11b and CD14.

## Discussion and conclusions

The natural history of leukemia in children with DS suggests that trisomy 21 directly contributes to the malignant transformation of hematopoietic cells. Approximately 15% of pediatric AML cases occur in DS children. Thus, ML-DS contribute more than 90% of the most common FAB subtype of DS AML patients and at the same time the majority of cases are diagnosed under the age of 4 years [15–17]. Zipursky et al. [9] have estimated that DS children have a 500-fold increased risk of developing ML-DS compared with non-DS children, highlighting the unique relationship between trisomy 21, leukemogenesis, and a specific leukemia phenotype. Other AML FAB subtypes have also been described in ML-DS including M0, M1/M2, and M6, but less frequently [15–17].

The cytogenetic profiles of ML-DS cases differ significantly from non-DS patients with AML [16, 18, 19]. ML-DS children show more frequently acquired trisomies of chromsomes 8, 11, and 19, dup(1p), del(6q), del(7p), dup(7q), and del(16q) [10]. Typically, the favorable translocations associated with non-DS AML [e.g., t(8;21); t(15;17); inv(16), 11q23 rearrangements] are rarely seen in ML-DS patients [10]. For ML-DS children older than 4 years cytogenetic features, molecular biology findings and response to therapy significantly diverge from younger patients, and are similar to the ones found in non-DS patients with AML [17]. However, recently de Souza et al. [20] reported a new ML-DS case associated with new acquired ACAs and they suggested those were clearly associated with the disease-progress and associated with an adverse risk. The case presented here share some feature with that of de Souza et al. [20] such as involvement of chromosomes 1 and 3 was and a poor outcome. Furthermore, chromosomal bands such as 1q25, 3q25, 16q24, and 20q13 are involved in chromosomal rearrangements frequently [21]. Moreover, translocations or inversions involving 3q21 and 3q26 are associated with a high-risk in AML, and these patients usually present with a poor prognosis [17]. However, in our case observed specific translocations der(16)t(1;16)(q25.3;q24) and t(3;20)(q25;q13.1) has never been reported as ACAs in ML-DS or AML cases to date [21].

Several studies have suggested that mutations in the hematopoietic zinc-finger transcription factor gene *GATA-1* (a transcription factor that regulates the differentiation of megakaryocyte and erythrocyte precursors), could be an initiating event in DS leukemogenesis [22, 23]. Besides the involvement of *GATA-1* and trisomy 21 is strongly associated with leukemogenesis [20]. Cytogenetic analyses revealed other acquired recurrent abnormalities associated with gain of chromosome 21. Forestier et al. [10] analyzed 189 ML-DS cases and they confirmed a distinct entity, originating from other genetic pathways than non-DS patients with AML.

Partial trisomy of chromosome 1q is commonly observed in infants with ML-DS and AMKL, which is most often resulting from an unbalanced translocation, like in the present case, or a simple duplication [24]. The long arm of chromosome 1 accommodates genes involved in the control of normal myeloid cell kinetics. Several interesting genes map in this region 1q, including *IL6RA* and *BCL2*-related are located at 1q21, *MNDA* (1q22), *CENPR* (1q32-q41), and *TP53BP2* (1q42.1~q42.2) [25].

For the chromosome 16 related imbalance two genes might specifically be considered: (i) Interferon regulatory factor 8 (IRF8) also known as interferon consensus sequence-binding protein located at 16q24.1, codes for a transcription factor, which plays a critical role in the regulation of lineage commitment and myeloid cell maturation including the checkpoint for a common myeloid progenitor to differentiate into a monocyte precursor cell [26]. (ii) The human *FOXF1* gene located at 16q24.1, previously denominated Forkhead Related ACtivator-1, encodes a homologue of the mouse forkhead box-F1 (*Foxf1*) transcription factor [27]. Gene knockout studies have shown that the function of mouse *Foxf1* is indispensable for organ morphogenesis, including the lung, liver, gallbladder, esophagus, and trachea [28]. Despite the largely unknown role of *FOXF1* in cancer, several lines of evidence have linked human *FOXF1* function to tumorigenesis [29]. Recently, it was suggested that *FOXF1* may play a dual role in tumorigenesis as an oncogene or a tumor suppressor gene depending on tissue cell types and disease stages [30].

As shortly discussed above, age has been recognized as a prognostic factor in ML-DS [31]. In fact, it has been proposed that DS children who present over 4 years of age are suffering from ‘normal sporadic AML’ occurring in a child with DS, rather than from ‘true’ ML-DS [32]. In addition, ML-DS patients with a history of transient myeloproliferative disease have a significantly better outcome than children with ML-DS without documented transient myeloproliferative disease [5]. Blink et al. [11] demonstrated that age ≥ 3 years and high WBC count (> 20 × 10^9^) are correlated with poor outcome (event-free survival) in ML-DS. These variables are also known from non-DS pediatric AML studies, in which older age and high WBC predict for poor outcome [33].

According to the literature the here observed partial monosomy 16q24 to 16qter has no clear impact on prognosis, and the meaning of the balanced translocation t(3;20)(q25;q13.1) needs to be delineated by further case studies. However, the present case of ML-DS may have an adverse outcome due to the partial trisomy 1q25.3 to 1qter, as also supported at least by one further similar case [20] and the known adverse effects of distal partial trisomy 1q in other malignancies [32].

## Material and methods

### Cytogenetics and molecular cytogenetics

Chromosomal analysis on peripheral blood sample using GTG-banding according to standard procedures [34] was performed prior blood transfusions. A minimum of 20 metaphase cells was analyzed. The karyotype was described according to the International System for Human Cytogenetic Nomenclature (ISCN 2016) [35].

Fluorescence in situ hybridization (FISH) using whole chromosome painting (WCP) probes for chromosomes 1, 3, 16, and 20 (MetaSystems, Altlussheim, Germany) was done according to manufacturer’s instructions [34]. Array-proven multicolor banding (aMCB) probes sets based on microdissection derived region-specific libraries for chromosomes 1, 3, 16, and 20 were hybridized and evaluated as previously reported [36]. A minimum of 10 metaphase spreads were analyzed, each, using a fluorescence microscope (AxioImager.Z1 mot, Carl Zeiss Ltd., Hertfordshire, UK) equipped with appropriate filter sets to discriminate between a maximum of five fluorochromes plus the counterstain DAPI (4′,6- diamino-2-phenylindole). Image capture and processing were performed using an ISIS imaging system (MetaSystems).

### Flow cytometric immunophenotype

Immunophenotyping was performed using a general panel of fluorescent antibodies against the following antigens typical for different cell lineages and cell types: CD1a, CD2, CD3, CD4, CD5, CD8, CD10, CD11b, CD11c, CD13, CD14, CD15, CD16, CD19, CD20, CD22, CD23, CD32, CD33, CD34, CD38, CD41a, CD45, CD56, CD57, CD64, CD103, CD117, CD123, CD138, CD209, CD235a and CD243; in addition antibodies to Kappa and Lambda light Chains, IgD, sIgM, and HLADr were tested. All antibodies were purchased from BD Biosciences. Samples were analyzed on a BD FACSCalibur™ flow cytometer. Autofluorescence, viability, and isotype controls were included. Flow cytometric data acquisition and analysis were conducted by BD Cellquest™ Pro software.

## Abbreviations

ACAs: Additional cytogenetic abnormalities
aMCB: Array-proven high-resolution multicolor banding
AMKL: Acute megakaryoblastic leukemia
AML: Acute myeloid leukemia
DAPI: 4′,6- diamino-2-phenylindole
D-FISH: Dual-color-fluorescence in situ hybridization
DS: Down syndrome
FAB: French–American–British classification
FISH: Fluorescence in situ hybridization
*Foxf1*: Mouse forkhead box-F1 transcription factor
Hgb: Hemoglobin level
*IRF8*: Interferon regulatory factor 8 gene
ISCN 2016: International System for Human Cytogenetic Nomenclature
ML-DS: Myeloid leukemia of Down syndrome
WBC: White blood cell count
WCP: Whole chromosome paint probes
WHO: World Health Organization classification
